# Operative Dentistry

**Published:** 1895-03

**Authors:** 


### MONTHLY DIGEST.

### Operative Dentistry.

DR. C. R. TAYLOR* writes of the "Treatment and Filling of Pulpless Teeth." He advocates a free opening into the pulp chamber and canals (sealing in ten per cent. sulphuric acid for a day or two, if necessary); thorough removal of all pulp tissue, using a smooth broach wound with a few fibres of cotton; washing out with three per cent. pyrozone; drying the canals and applying eucalyptus oil filling with chloro-percha, in which is inserted gutta-percha points, or a fine copper point, after evaporation of the chloroform. The oil of eucalyptus causes the gutta-percha to adhere to the dry walls of the canals, and the metallic point squeezes out the excess of gutta-percha.

### CERVICAL CAVITIES.

Dr. WM. L. FISH presented the Odontological Society of Pennsylvania* with a new clamp for pushing back the gum from cervical cavities, so constructed that a single clamp serves for any tooth in the mouth, and which being attached to the tooth adjoining the one to be operated upon, leaves a clear space for operating. It is operated by the simple turning of a thumb-screw. Dr. Fish gave in detail his method of preparing and filling these cavities. He denounces the retaining-point as "an abomination, a delusion and a snare," preferring a slight undercut, made with inverted cone bur, thus shaping the largest diameter of the cavity at the bottom. After making the undercut, he levels the edge of the cavity from within outward, using a diamond cone point. He then proceeds to line the cavity with absolutely soft gold, followed by semi-cohesive worked in by hand-pressure with a rotary motion. He then fills with cohesive gold.

For the soft or plastic filling, he discards the beveled edge and makes the cavity as near as possible parallel through the thickness

---

* Dental Review, November 15, 1894.
* International Dental Journal, December, 1894.

of the enamel. In the manipulation of alloys he prefers ball instruments, which work the mercury to the surface, working down any surplus with the burnisher, that no mercury may remain in the filling.

In the discussion, Dr. Truman thought the use of this clamp would create such irritation that pathogenic germs would gather round the tooth, resulting in inflammation of the pericementum, if not pyorrhœa alveolaris.

Dr. Faught thinks that for those who use clamps, this one will do the work better than any of those now furnished by the depots.

Dr. Truman and Boice took issue with Dr. Fish on the subject of retaining points, while Drs. Brown and Faught agreed with the essayist.

### SULPHURIC ACID IN ROOT CANALS.

Dr. J. R. Callahan read before the New Jersey State Dental Society* a "Second Paper" on the use of sulphuric acid for opening up root canals.

The acid, in a forty or fifty per cent. solution, is applied directly upon and about the dead pulp by means of a broach, bent to a right angle, with a little cotton twisted on the end. Old, discarded broaches, can not be used for this purpose, as the same broach should not be used a second time, the acid making them very brittle. The acid, by a process of dehydration, causes the pulp to shrink and toughen so that it is easily removed ; a drop of the solution is then placed over the mouths of the canals, it being sometimes necessary to sink a little depression to keep the acid where it is wanted. With a number five Donaldson nerve-canal cleaner, bent to a suitable angle, with the shank cut short to make it fit close to the handle, rigid and strong—enter the canal slowly and carefully, with a pumping motion, the acid preceding and following the broach closely, destroying all septic matter with which it comes in contact. When all the canals have been thus treated, with a Dunn syringe fill the cavity with a saturated solution of bicarbonate of soda, which, by contact with the acid, lib-

* Dental Cosmos, December, 1894, erates carbonic acid gas, causing an effervesence which carries all the broken up tooth and pulp substance out of the canals, out of the tooth, on to the rubber dam, leaving a deposit of bicarbonate of soda lining the whole tooth. When this is removed, the canals are found white and clean. If desired to enlarge the canals, repeat the operation. The canals being thus thoroughly opened, thoroughly cleaned and thoroughly aseptic, need only to be thoroughly dried when they can usually be filled at once. The action of the sulphuric acid is self-limiting, it is a pronounced germicide It acts with greatest vigor on diseased tissue, breaking it down and destroying it, leaving a fresh aseptic surface, in a fistulous tract as well as in the root. By its action, the dentine is softened for an indefinitely short distance, which being removed by the broach, the surface is again attacked as before till the desired limit is attained, but no further. The method is desirable because of its ease, brevity, thoroughness, and permanent success when intelligently used, and is applicable wherever the pulp is dead, whether recently or for a long time, fresh or putrescent, with or without abscess, blind or fistulous.

At the same meeting, Dr. C. N. Johnson read a paper on the management of "Pulpless Teeth"*. For the destruction of the pulp, Dr. Johnson prefers cobalt to arsenious acid, as being less liable to cause pain. The cobalt is applied directly to the pulp on a small pellet of cotton moistened with one of the essential oils and sealed in, oxyphosphate flowed over the cotton. In deep approximal cavities the gum is protected before applying the cobalt by building a barrier over the gum septium and along the gingival wall of the cavity to near the point of pulp exposure. In the anterior teeth the cobalt is removed after twenty-four hours, because of possible discoloration. In molars and bicuspids, it is left undisturbed for one week. When the cement is removed from the anterior teeth, the cobalt is washed out with one of the essential oils, the cavity dried, and a pellet saturated with the oil sealed in for one week.

At the second sitting for the posterior teeth (after one week),

* Dental Cosmos, December, 1894.

the rubber dam is applied, the cement removed, the cobalt washed out by flooding the cavity with absolute alcohol, evaporating till the cavity is dry. A sharp bur is used to open up the pulp chamber, lacerating and cutting up the body of the pulp till all the debris can be washed out with alcohol, which also assists in drying out all moisture in the pulp tissue in the root canals, facilitating its removal. The canals are then flooded with an antiseptic, wiped out with absorbant cotton on a broach; the canal is again flooded with alcohol and dried, and it is ready for immediate filling.

When pulps are found dead, but without fistulous opening, the rubber dam should be applied before drilling into the tooth with a non-irritating, non-effervescing antiseptic, at hand for im mediate use the moment the pulp chamber is penetrated, the greatest care being taken not to force any septic matter through into the apical space. With absorbent cotton or bibulous paper, and repeated flooding with the antiseptic, the greatest part of putrescent matter may be removed without causing any disturbance at the apical foramen.

The canals are then flooded with alcohol and evaporated to dryness, then flooded with the antiseptic, a loose pledget of cotton saturated with the same, placed loosely in the chamber, and the whole sealed in for a week. If no trouble ensues, the canals may then be again washed out with alcohol, dried, flooded with an antiseptic, dried again and filled.

In case of fistulous opening, after removal of all putrescent matter and cleaning the canals, the antiseptic (usually Black's 1, 2, 3), should be injected through the canal till it appears at the opening on the gum. This may be effected by saturating cotton with the medicine, packing it in the canal, and forcing down upon it a plug of rubber such as is used for vulcanizing. When free passage is secured, pack in freshly saturated cotton, seal in and dismiss for a week. If the fistula is then found closed, the roots may be filled. If the fistula is persistent, carbolic acid, ninety-five per cent., may be injected; or if the opening is free, it may be preceded by pyrozone, three per cent., and the case dismissed for two weeks. Frequent treatments are to be avoided, unless there is a copious flow of pus. In chronic cases of slight discharge, after the third treatment, the dressing in the canal should be left undisturbed for a month, though in the meantime the fistula may be treated through the opening in the gum.

For filling canals, Dr. Johnson prefers gutta-percha points, using as a lubricant gutta-percha dissolved in eucalyptol, as being less irritating to the apical tissues than chloroform, with consequent less soreness after filling.

### ELECTRO-DEPOSIT FILLINGS.

DR. SIGEL ROUSH* portrays, as a possibility in tooth-filling, the ideal advantages offered by applying the principle of electro plating to the filling of cavities in human teeth. The deposit formed by this process is so close, the microscopic particles so penetrating, the adaptation so perfect, that a cavity filled by such a process would be nothing short of perfection. While admitting that his experiments thus far have been conducted on teeth out of the mouth, in which he has obtained some most perfect fillings, it is his firm belief that the difficulties to be overcome in filling teeth *in situ* by this process are not insurmountable, and that in this way we will yet produce the truly ideal gold filling.

### GOLD CROWNS.

At the annual meeting of the Minnesota State Society,† Dr. W. P. Dickinson gave a demonstration upon the formation of the occluding surface of biscupid and molar gold crowns, emphasizing the importance of securing correct articulation, permitting utilization of the entire surface-area in mastication, as with the natural teeth.

### PORCELAIN CROWNS.

DR. HENRY A. KNIGHT presented the Minnesota State Dental Society‡ with a combination porcelain crowns, and demonstrated its value. He claims for his crown that it combines all the advantages of both the Richmond and the Logan crown, without the disadvantages of either.

* Dental Cosmos, December, 1894.

† Dental Review, November 15, 1894.

‡ Dental Review.

### BRIDGEWORK.

Dr. Van Woert, in the New Jersey State Society,* gave a "Blackboard Chalk-talk" on his system of dovetail backing and removeable faces in bridgework, describing a method of making the necessary instruments and appliances from cast-off instruments. He also illustrated various uses of common tin or soft-solder in prosthetic dentistry. In making partial plates, after adjusting the backings to the teeth, he removes them, having left the pins perfectly straight, and solders the backings to the plate with gold as usual. He then drills holes the size of the pins, covers the sockets and pins with a solution of zinc chlorid, readjusts the teeth and attaches them in place with soft-solder by means of a soldering iron. This is quickly done, and admits of easy repair.

In crown work, to secure a very close adaptation of dowel to root-canal in badly decayed roots, he inserts a small wire in the prepared canal and packs gutta-percha around it, withdrawing it when cool by means of the inverted wire. This cone is invested in equal parts plaster and marble dust, giving a mold the exact form of the canal. When dried and heated sufficiently to fuse ordinary tin solder with which it is filled, the selected Logan, or other dowel crown, is coated with sweet oil, the pin coated with a solution of chlorid of zinc, heated and inserted into the mold containing the soft-solder and cooled immediately. Union between pin and solder gives an exact fitting dowel, which can be inserted in the root canal with so little cement that there can be heating of the pin to cause expansion and consequent shrinkage, and therefore no ingress of fluids, softening of phosphates and loosening of the crown. A more perfect joint is made in this manner than is possible with cement or plastics, and is considered by Dr. Van Woert as preferable even to the How anchor screw with amalgam.

### CARE OF INSTRUMENTS.

Dr. Wm. H. Potter† uses the Arnold sterilizer, in which a metal chamber is kept at 212° F. for the sterilzation of all dental

* Cosmos, December, 1894.

† International Dental Journal, December, 1894.

instruments. Nickel-plated instruments are simply exposed in the moist heat. Steel instruments are placed in a zinc tray containing carbonate of soda. In his ordinary piston syringes, he substitutes a felt packing, touched with soap for the greased packing, which the heat would ruin. Syringes thus fortified are put through the sterilizer after each operation. His hypodermic syringes are fitted out with a wash-leather packing, soaked in hot mutton tallow. The packing is removed after each operation and all parts boiled in a soda solution. Rubber dam straps and holders are sterilized after each case.

Separate labeled mouth-mirrors are a necessity from the impossibility of anything more than a washing. In the discussion of this paper by the Hartford Odontological Society, the necessity of the most thorough sterilization was strongly emphasized, and especially careful washing with soap and water before sterilization. Dr. Cooke washes his hand-pieces, etc., and puts in a weak solution of bichloride. Dr. Taylor unscrews the cone-socket of his hand piece, sterilizes the point, and washes the rest as best he can. It is impossible to perfectly sterilize the mouth, but the rubber dam protects the soft tissues, and the hard tissues are not very susceptible to the action of germs.

In reply to certain statements made by Dr. Bromell, in an article entitled "Metallic *versus* the Plastic Base for Artificial Dentures," (See *International Dental Journal*, October, 1894), Dr. Wm. Truman asks the question: "Is Prosthetic Dentistry Lagging?"* with a very decided negative reply.

Dr. Truman classes the statements so frequently made to this effect among the stock "facts" used in dental meetings without any question as to their foundation, and as merely evidencing lack of study and thoughtful sifting of evidence. Judging the progress of dental science by its ability to make happier and more pleasant the life of each individual of the community in so far as their pleasure and happiness can be enhanced by dental art, he considers that as the use of vulcanite enables thousands of persons of limited means to enjoy the comfort and satisfaction of artificial dentures, who otherwise would be constrained to do without them,

* International Dental Journal, December, 1894.

therefore the use of this material is by no means the unmitigated evil it is so often made to appear, neither was its introduction "the nucleus from which sprang dental quackery." While operative dentistry is more and more successfully accomplishing its mission of preserving in comfortable usefulness the natural organs, it still remains—with its expensive materials and expensive methods—a luxury the well-to-do alone can afford, while competition has so reduced the cost and improved the quality of artificial dentures that to-day few, if any, for financial reasons, need do without them. If service to the community, therefore, be the test, it is not prosthetic dentistry that is lagging in the race.

PROSTHETIC DENTISTRY.

DR. M. L. FAY read a paper before the Union Convention of the Sixth, Seventh and Eighth District Dental Society of New York*, on the Preparation of the Mouth—what to extract and what to leave—the four staple materials for impressions—plaster, beeswax, gutta-percha, and modeling compound—the conditions governing the choice of material, and how to take an impression. He was followed by Dr. H. B. Meade, on Taking the Bite—the Selection and Arrangement of Teeth—Restoration of Contour and Waxing up the Case. Dr. Geo. B. Snow followed with a paper on Flasking, Vulcanizing and Finishing Rubber Plates, going very minutely into the details of the various steps of the work. These papers were discussed as a whole, at considerable length. Lack of space prevents a reproduction.

No. 1, Vol. I., of the *Dental Digest*, the official organ of the "Dental Protective Association" of the United States, opens with "Suggestions on Developing and Conducting a Dental Practice on Business Principles,"† by Dr. I. N. Crouse.

The desirable qualifications for a dentist are thus enumerated. "A liberal education, manipulative skill, artistic taste, business tact, industry, perseverance, good judgment, even temper, self-control, enthusiasm, and last, but not least, strict integrity." He

* Dental Cosmos, December, 1894.

† Dental Digest, January, 1895.

asks: "Is it too much to say that he needs all the logic of the lawyer, the scientific knowledge of the physician, and the high moral ideas and sense of responsibility of the clergyman, combined?" The selection of an office and its equipment is next considered. How to make patients appreciate the services you render, and some of the most advantageous methods of operating, will be considered in the next issue.

PROGRESS IN OPERATIVE DENTISTRY.

Dr. Thos. E. Weeks, Minnesota State Dental Society,* in pursuance of a previous resolution adopted by the Society, presented a report on the "Progress made in Operative Dentistry" during the past year, as gathered from current literature.

A step in advance noted by him is the increasing tendency towards the recognition of *principles* rather than *methods.* While correct methods are indispensable, a thorough understanding of the underlying principles is essential to the attainment of perfection. If a condition is to be remedied, the causes responsible for the condition must be recognized. The characteristics of materials used and instruments employed, come under the law of observance of principles. Of special importance is the recognition of the principles governing *contact, contour* and *occlusion* in filling teeth, contouring both for form and for contact, restoring ideal conditions to the fullest extent possible. The principles governing the use of gold as a filling material, and the causes of failure, new methods in the use of amalgam, new cements, "combination-filling," the renaissance of tin, and the marked advance in the treatment of root-canals, all received due attention, both in the paper and the discussion which followed the reading. In the discussion, Dr. Knight emphasized the value of the combination of amalgam and cement, suggested by Dr. St. John, using a thin layer of cement next to the tooth walls, followed by a mixture of amalgam and cement for the body of the filling, finishing the surface with amalgam. The mixed amalgam and cement is soft and adhesive, sticking to the sides of the cavity, so that it can be

* Dental Review, December 15, 1894.

placed in any cavity, requiring less positive retaining pits than other methods.

### A REVIEW OF NEW REMEDIES.

In the Minnesota State Dental Society,* Dr. O. A. Weiss read a report on various remedies of recent introduction, or new uses of well-known medicaments, including potassium permanganate as an antidote for opium poisoning, the uses of pyrozone, sodium peroxide, kalium and natrium, sulphuric acid for opening up root-canals, salol as a root-canal filling and pulp-capping, and cocaine *pro.* and *con.*

In the discussion of the paper, Dr. Van. Woert added to the list *vinaigre de pennes*, introduced by Dr. Bogue, as a valuable antiseptic, trichloracetic acid, alumnol and phytolacea. Very satisfactory results were reported from the use of trichloracetic acid and pyrozone. Kalium-natrium and sodium peroxide do not seem to have given general satisfaction, probably from the great caution requisite in their use.

### ELECTRO-THERAPEUTICS.

Dr. M. G. Jenison read a paper on this subject before the Minnesota State Dental Society.† After showing the medicinal and technical difficulties to be overcome, and the thorough special education necessary to make this valuable adjunct available to the dental practitioner, he enumerates its practical applications—as a local stimulant, a constitutional tonic, in the treatment of neuralgic affections, in acute and chronic inflammations, in obstinate cases of hemorrhage, as an anæsthetic, and as a germicide. With these possibilities open before us, it is well worth our while to at least investigate the subject thoroughly.

### HEATING AND ANNEALING APPARATUS.

Dr. Henry F. Sibley exhibited before the American Academy of Dental Science, Philadelphia,‡ an apparatus ingeniously contrived to heat over an ordinary alcohol lamp or Bunsen burner,

* Dental Review, December 15, 1894.
† Dental Review, December 15, 1894.
‡ International Dental Journal, January, 1895.

a sand bath containing a receptacle for the hot-air chamber of the chip-blower; a water-reservoir with an aperature for the point of the water syringe, the metal top of which serves as a gutta-percha-heater; it also carries an annealing tray, and on one side is a ring which supports a bottle of obtundent. The apparatus, which is provided with a thermometer, is compact, and arranged upon a base sufficiently heavy to give a firm support to the different parts, which swing around upon standards, being brought into use as required.

THE RELATIVE PENETRATING POWER OF COAGULANTS.

Dr. Jas. Truman read a paper on this subject before the Academy of Stomatology, Philadelphia.* Admitting as beyond question the importance of reaching the microscopic contents of the dentinal tubuli preventing decomposition and septic emanations, and recognizing the difficulties of manipulation, the desired result can only be attained through coagulation, or through the diffusability of various essential oils, aided by osmotic action, both methods having decided advocacy, and probably both having a positive value, the extent of which has yet to be determined. After giving a brief resume of the views of Drs. Black, Harlan, Hugenschmidt, Kirk, and others, and reaffirming his own position, taken in 1889, that "coagulants placed in the central canal would permeate the tubuli and coagulate the contents," Dr. Truman proceeds to give the results of a series of experiments which have occupied several months, with repetitions under varying conditions, demonstrating the correctness of the conclusions reached, namely, that coagulants will penetrate tubes of the minutest character possible to handle, the penetration being independent of circulation and in opposition to gravitation. The purpose of the article is to show, through the results of these experiments, that the belief that *coagulation furnishes its own barrier to diffusion*, is based on an error of observation. The details of this series of experiments, with a series of tubes numbered from one to fifty, can not be given here. Two conclusions were reached, *viz*, that "coagulants do not prevent by their own action diffu-

* International Dental Journal, and the Dental Cosmos, January, 1895.

sion throughout the entire tube," and that, "in proportion to the coagulating power of the agent, will be the penetrating force, independent of gravitation."

In the discussion, several of the speakers expressed a desire for further study of the paper before pronouncing upon the value of the experiments. Dr. Harlan stated that he would present the other side of the question before the First District Dental Society of New York at the January meeting.

### TREATMENT OF PYORRHŒA ALVEOLARIS.

Dr. W. X. Sudduth, in the Minnesota State Dental Society,* read a paper on this subject, preceding it by an abstract of an earlier paper, in which the writer took the ground that pyorrhœa alveolaris is a local affection, depending usually upon a general catarrhal dyscrasia for its ability to engraft itself upon the tissues, being essentially catarrhal in its nature, beginning as a simple gingivitis and extending to the pericementum. Numerous predisposing causes were enumerated, the exciting cause being infection from the fluids of the mouth, experiments having demonstrated the practice of lactic acid in the saliva in mouths in which pyorrhœa alveolaris was present. Gout is regarded by the author as simply a complication, and not an etiological factor. The treatment advocated consists in the removal of all local causes of irritation, fixing firmly all loose teeth, correcting false articulation, and the use of palliative mouth washes and local stimulating antiseptics. The three per cent. pyrozone is used as a mouth wash, the soapy taste overcome by dropping a soda-mint tablet in the proper quantity just before using. This increases the antiseptic action and neutralizes the small degree of acidity present. In obstinate cases the pockets may be packed with a salve of laudlim and sozo-iodol and zinc. In case of infrequent dressings, pack with beeswax (softened in hot water), to which sulphate of zinc is added. This will retain its form and antiseptic qualities many days, being forced out of the pockets as healing progresses from the bottom. In the discussion, Dr. G. V. I. Brown emphasized the importance of correcting mal-occlusion.

* Dental Review, December 15, 1894.

In very persistent cases he has found it necessary to devitalize the pulp before checking the pus flow.

### FACIAL NEURALGIA FROM A DENTO-SURGICAL STANDPOINT.

Dr. M. H. Cryer read, before the New Jersey State Dental Society,* a paper on this subject, giving a *resume* of the local anatomy of the fifth pair of nerves, with a view to an understanding of the aberrations of function; a list of the more frequent causes of facial neuralgia, with comparative illustrations, emphasizing the importance and difficulties of diagnosis.

Being convinced, through the process of exclusion, that the case is one of neuralgia, the same process should be followed in determining the cause, while all external and constitutional causes should be excluded before the surgeon is justified in opening the face or brain case for a resection or a removal of the Gasserian ganglion. The details of several cases were given.

### ORAL SURGERY.

Dr. Chas. A. Van Duzee, in the Minnesota State Dental Society,† read a paper on this subject, taking the ground that the dental practitioner betrays a sacred trust if not competent to diagnose the pathological condition of the entire locality, though the operative skill necessary in the oral surgeon can not be attained without special training and clinical work, requiring more time than is usually accessible for the student of dentistry. Oral surgery is dependent, among other things, upon a thorough knowledge of physiology, anatomy, histology, and pathology, together with a ripe and practical acquaintance with human nature. When, through our knowledge, we diagnose a lesion that requires systemic treatment or surgical interference, if we call in the proper assistance, go to those whose daily work requires the equipment and manipulative skill which such cases must have, we shall have done our duty to the patient, to ourselves, and to our profession. In the discussion of the paper, Dr. Weeks said that the essential features were *honesty*, *ability* and *thoroughness*; honesty

* Cosmos, January, 1895.
† Dental Review, December 15, 1894.

with ourselves, our patients, and our Maker; ability, including knowledge—anatomical, physiological, and pathological; thoroughness in getting at the bottom cause in the first instance, not temporizing and letting the disease get ahead of the surgeon instead of *vice versa.*

### A NEW METHOD OF APPLYING FORCE IN THE REGULATION OF TEETH.

Dr. Edward H. Angle* describes a novel way of exerting force to a moving tooth, the power being derived from lengthening a properly applied wire by pinching, or compressing it laterally, between suitably formed breaks of strong pliers. In the cases illustrated the wire (preferably the "anchor and retaining wire G" of the angle system); being applied and secured to the teeth by the angle methods, is gradually lengthened by "pinching," as described, at varying intervals of time, each pinch lengthening it about 1-100 of an inch, two or three "pinches" each day or alternate day, or once a week in some cases, moving a tooth as desired. The wire is finally left in position as a retaining appliance, being cleanly, compact and efficient.

### THE DELIVERY OF FORCE BY PLUGGER POINTS.

Dr. E. A. Royce, in a paper read before the Odontological Society, Chicago,† gave additional arguments and evidences of the correctness of his published conclusions in regard to the behavior of gold under the plugger point.

His paper was an elucidation of the laws of devotion, especially the law for the wedge, as illustrated by the movement of boats in the water, force delivered to marbles, bullets or dice, the spreading of a rivet under the round-faced hammer, etc., all tending to prove that *gold does spread* during the process of condensation, provided that an oval-faced plugger be used, the direction of the force depending, not upon the line of impetus, but directly upon the shape of the face that delivers the force. He very pertinently asks: "If force is delivered by an oval-faced

* Dental Digest, January, 1895.
† Dental Review, December 15, 1894.

point, at an angle to the line of impetus, and that force is sufficient to change the position of the particles of gold, if they are not driven to one side, or in a direction more or less toward the walls of the cavity, where do they go, and what is the mechanical law that governs their movements?" To secure the perfect working of gold over the margins of the cavity the spreading force is absolutely indispensable, and the only way in which this force can be obtained is by the use of instruments, the outlines of which are based upon scientific principles.

### FILLING ROOT CANALS.

Dr. I. C. St. John, in the Minnesota State Dental Society,* after reviewing various proposed methods of filling root-canals, gives his own, as follows: A solution of base-plate wax in chloroform or eucalyptus is used for saturating the canal walls, canal points of the same being used instead of gutta-percha points. These points are first chilled and then carried into the canals with cold instruments; the mass of wax is then penetrated with a heated root-canal drier melting the wax and more perfectly adapting it to the walls of the canal and the tubuli. For further condensing the filling, a ball of cotton may be placed over the mouths of the canals and forced down. Experiments out of the mouth have demonstrated the space apparently perfectly filled by this mothod.

Dr. Jas. H. Daly, American Academy of Dental Science,† offered a method of filling root-canals, partly in reply to an article by Dr. Nelson Shields, advocating gold-foil as the only material for this purpose. The important feature in filling root-canals being to thoroughly seal the apex of the root, without forcing everything through. Dr. Daly obtains the best results by using a tapering gold wire or broach, sharpening the extreme point so that the instant it passes the apex the patient will give warning. The length of the canal being thus known, is marked upon the wire, which is withdrawn, the tiny sharp point filed off, and the wire notched at about one-sixteenth of an inch from the end. It

* Dental Review, December 15, 1894.

† International Dental Journal, January, 1895.

is then driven home to the apex, a slight twitch breaking the wire off at the notch, leaving the apex securely sealed with a "Royal" metal.

In the discussion, Dr. Williams advocated tin or lead points, as having more conservative qualities than gold. Whatever he uses is well coated with some antiseptic paste. He leaves an end projecting into the pulp-chambers, so that it can be withdrawn in case of subsequent trouble.

### PULP DEVITALIZATION.

Dr. Daly's paper on Root-Filling gave rise to an incidental discussion of pulp devitalization, a painless method being a great desideratum.

Dr. Daly prefers Dr. Harlan's paste, *viz.*, arsenious acid and hydrochlorate of cocaine equal parts triturated with lanoline; this makes a paste easy to handle and painless in its effects. Dr. Fillebrown would invariably reduce the inflammation and quiet the pain before applying arsenic, and prefers Prof. Flagg's method —arsenious acid and creosote diffused through finely chopped cotton fibres. A positive method, not followed by unpleasant features, is the administration of gas and forcible removal of the pulp, with a barbed broach, but patients will not always permit this.

### PREPARATION AND FILLING OF PROXIMATE CAVITIES IN BICUSPIDS AND MOLARS.

Dr. E. H. Allen read a paper on this subject before the Wiscon State Dental Society,* with the view of formulating a system with general rules. He emphasizes the importance of obtaining sufficient space (giving preference to the wedge over separators), and of waiting till all soreness has passed away before operating. Then adjust the rubber dam and open through the occlusal surface to get easy access and good light; the boundary of the cavity should be extended until tooth-structure can not come in contact after the teeth resume their normal position; side walls should be nearly parallel; leave no overhanging edges of enamel; cut

* Dental Review, December 15, 1894.

out the fissure across the occlusal surface, and if extensive cavities on both proximate surfaces, unite them across the occlusal surface; have all accessible margins carefully beveled (for this he uses a No. 50 stone-cut bur made to special order).

Having given full details of his rules for preparing cavities, the material for use in filling is next considered, gold being given the preference, unless when the cavity is so large as to nearly approach crown-work, or where the rubber dam can not be applied; also where the operator is doubtful of his ability to properly reach every part of the cavity. For the upper teeth, his preference is for cohesive gold in ribbons, condensed with Bonwill's mechanical mallet. For the distal surface of lower teeth, he uses a matrix and gold in soft pellets. He emphasizes the importance of thoroughly condensing the gold against all parts of the cavity, and of restoring the natural contour of the tooth so that approximal fillings will knuckle together near the occlusal surface. The same care is necessary in preparing cavities and in furnishing fillings with amalgam as with gold.

### RESTORATION OF BROKEN MOLAR.

Dr. Noyes, in the Chicago Dental Society,* described what he termed a "make-shift operation," by which he restored a badly broken lower second molar, of which nearly the whole body of the crown was gone, being broken about half-way to the gum through the lingual two-thirds of the mesial and the anterior third of the lingual wall; buccally it was decayed across the whole length, and there was a bucco-lingual split either through, or taking off the anterior root. The patient was not willing to have the tooth cut off and crowned. A good-sized platinum wire was placed around the tooth, the ends twisted tightly, the ends and the portion across the buccal-wall falling within the cavity to be enclosed in the subsequent filling. The wire passed through a notch at the bucco-lingual angle preventing it from working down into the gum. The crown was then restored with amalgam, the filling holding the wire, and the wire holding the filling.

* Dental Review, December 15, 1894.

[*To be Continued.*]

